# Association between sleep quality and psychological wellbeing in 175 elite adult athletes: a cross-sectional study

**DOI:** 10.3389/fpubh.2025.1681005

**Published:** 2026-01-12

**Authors:** Hanyu Li, Heng Liu

**Affiliations:** 1 Faculty of History and Culture, Chengdu Sport University, Chengdu, China; 2 College of Physical Education, Chongqing University, Chongqing, China

**Keywords:** elite athletes, sleep quality, mood disturbance, fatigue, PSQI

## Abstract

**Purpose:**

This study examined the relationship between sleep quality and mood state and identified psychological predictors of sleep disturbance in elite adult athletes.

**Methods:**

A stratified cluster sample of 175 elite adult athletes (67 males, 108 females; age 22.6 ± 3.7 yr) was recruited from Sichuan Province, China. Participants with a Pittsburgh Sleep Quality Index (PSQI) score ≥ 5 were classified as having disturbed sleep (*n* = 87); those scoring < 5 were assigned to the normal-sleep group (*n* = 88). Sleep quality was assessed with the PSQI; mood state was evaluated with the Profile of Mood States (POMS).

**Results:**

The disturbed-sleep group scored significantly higher than the normal-sleep group on the PSQI global score and on all seven component scales (*p* < 0.01). Tension, anger, fatigue, depression, confusion, self-esteem and total mood disturbance (TMD) were also markedly elevated in the disturbed-sleep group (*p* < 0.01). Pearson correlations revealed positive associations between PSQI global score and both fatigue (R = 0.242) and TMD (R = 0.347) (*p* < 0.01). Multiple linear regression indicated that fatigue (*β* = 0.581, *p* < 0.001) and total mood disturbance (TMD, *β* = 0.218, *p* = 0.004) were independent predictors of PSQI global score, explaining 57% of the variance (adjusted R^2^ = 0.57, *F*(2,172) = 115.59, *p* < 0.001). Sex-stratified analysis indicated that the correlations between fatigue, TMD and sleep quality were significantly stronger in female athletes (R = 0.368 for fatigue, R = 0.402 for TMD) than in male athletes (R = 0.286 for fatigue, R = 0.312 for TMD), and depression scores in the disturbed-sleep group were significantly higher in females than in males (*p* = 0.008).

**Conclusion:**

Sleep disturbance in elite athletes is closely associated with psychological fatigue and overall mood disturbance, with fatigue as the core predictor. Significant gender differences exist: the mood-sleep association is stronger in female athletes, and sleep disturbance has a more prominent impact on their depressive mood, requiring targeted interventions.

## Introduction

Sleep underpins recovery, neural plasticity and the regulation of cognition and emotion (1, 2); conversely, even brief deprivation down-regulates hippocampal tight-junction proteins and triggers neuronal apoptosis in animals (3, 4), while persistent disturbance—via metabolic dysregulation, hypothalamic–pituitary–adrenal hyper-activation and neurotransmitter imbalance—elevates the risk of anxiety, depression and subjective fatigue in humans (5, 6). Paradoxically, although regular exercise is widely assumed to improve sleep (7, 8) and psychological wellbeing (9, 10), elite athletes report more sleep problems than the general population because chronic high-load training and competitive stress override the protective effects of physical activity (11, 12). This athlete-specific contradiction underscores the need to examine sleep–psychology links separately from the healthy-exerciser model.

Current athlete-centered research has concentrated on competitive anxiety (13), burnout (14) and emotion-regulation deficits (15), typically treating sleep as an outcome rather than a mechanistic variable. Longitudinal and experimental evidence indicates a bidirectional loop: emotional distress forecasts deteriorating sleep, whereas sleep loss amplifies emotional lability and, by impairing attentional control, ultimately compromises performance (16). Yet cross-sectional data in elite adult athletes remain scarce, especially studies that simultaneously apply standardized instruments for both sleep quality and multidimensional mood states. Moreover, existing work has focused on Western cohorts (17–19), leaving sleep–psychology interactions in East Asian athletes largely unexplored. These gaps generate two key questions: (1) What is the strength and profile of the association between sleep disturbance and emotional distress in high-level adult athletes? (2) Do athletes with different sleep profiles differ significantly in mood state, thereby offering precise intervention targets?

Using the Pittsburgh Sleep Quality Index (PSQI) and the Profile of Mood States (POMS), the present cross-sectional study recruited elite adult athletes from Sichuan Province, China, to provide an evidence base for targeted, combined psychological-and-sleep interventions in sport settings. This study hypothesized that fatigue, depression and tension would be positively associated with PSQI-defined sleep disturbance.

## Materials and methods

### Participants

This cross-sectional study was conducted between May and June 2025 in Sichuan Province, China. Stratified cluster sampling was used to recruit elite adult athletes, with 1:1 matching between disturbed-sleep and normal-sleep groups based on sex, age (±1 yr), sport category (power, endurance, skill, team), and training years (±1 yr). The Pittsburgh Sleep Quality Index (PSQI) was used to classify participants into disturbed-sleep (PSQI ≥ 5) and normal-sleep (PSQI < 5) groups.

Initially, 580 athletes from 20 provincial squads were approached. Inclusion criteria: age 18–30 yr.; national first-class athlete status or above (i.e., having finished in the top 32 in an individual national championship or top 16 in a team event within the past year); ≥ 5 yr. of systematic training; and ≥ 20 h sport-specific training per week during the preceding 4 weeks. Exclusion criteria: acute sports injury or medical condition precluding training in the past 4 weeks; diagnosed sleep disorder, depression or other psychiatric condition currently treated with medication; questionnaire completion time < 5 min; or > 10% missing data. After electronic screening via the Wenjuanxing platform, 552 valid questionnaires were retained, including 102 with PSQI ≥ 5. This study defined PSQI ≥ 5 as “disturbed sleep” and PSQI < 5 as “normal sleep” (20). Ninety athletes were randomly selected from each group (disturbed-sleep and normal-sleep) for matching. Five participants were excluded due to > 10% missing POMS data, resulting in a final sample of 175 (disturbed-sleep: *n* = 87, 32 males/55 females, age 22.3 ± 3.6 yr., training 7.6 ± 2.6 yr.; normal-sleep: *n* = 88, 35 males/53 females, age 22.9 ± 3.9 yr., training 7.7 ± 3.2 yr). Matching variables showed no significant between-group differences (*p* > 0.05). This study was approved by the Human Research Ethics Committee of Chengdu Sport University (approval number: 202525) and conducted in accordance with the Declaration of Helsinki. All participants provided written informed consent.

### Questionnaire survey

Testing was scheduled on non-training days or ≥ 2 h post-session, with no competition that evening. Two trained postgraduate sport-psychology students administered paper questionnaires anonymously; completion time was capped at 15 min. The paper and electronic versions contained identical item wording and order.

Pittsburgh Sleep Quality Index (PSQI): 18 self-rated items yielding seven component scores (range 0–21). Higher scores indicate poorer sleep. Chinese-version reliability: 0.65–0.84; validity > 0.85 (21).

Profile of Mood States (POMS): 40 items rated 0–4 (from “not at all” to “extremely”) assessing tension, anger, fatigue, depression, vigor, confusion and self-esteem. Vigor and self-esteem are positive; the remaining five are negative. Total mood disturbance (TMD) = (sum of negative scores) – (sum of positive scores) + 100. Higher TMD indicates poorer mood. Chinese-version reliability: 0.60–0.82; validity > 0.90 (22).

### Confirmatory validity of questionnaire

**Figure 1:**
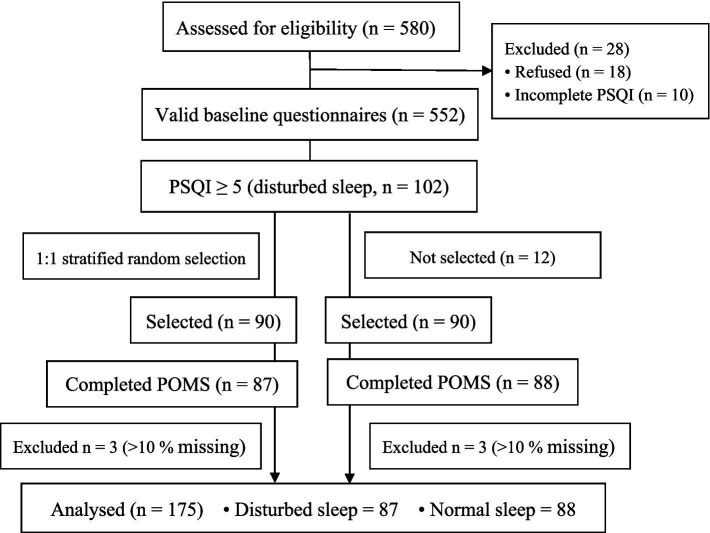
Flow of participants through the study. PSQI, Pittsburgh Sleep Quality Index; POMS, Profile of Mood States. A 1:1 matched sample was achieved by stratified random selection based on sex, age (±1 year), sport category, and training years (±1 year).

Cronbach’s *α* across 175 valid protocols was 0.77; test–retest reliability (ICC) in a random 20% subsample after 7 days was 0.82. Confirmatory factor analysis yielded acceptable fit: χ^2^/df = 2.06, CFI = 0.94, RMSEA = 0.05 (Figure 1).

### Statistical analysis

Analyses were performed in SPSS 26.0. Normality was confirmed (Shapiro–Wilk). Descriptive statistics are presented as mean ± SD. Between-group differences in PSQI and POMS scores were examined with independent-samples *t*-tests. Pearson correlations assessed associations between PSQI global score and POMS dimensions. A-priori forced-entry multiple linear regression was conducted with PSQI global score as the dependent variable; fatigue and total mood disturbance were entered simultaneously based on theoretical grounds and bivariate correlations (23). Significance was set at *α* = 0.05.

## Results

Compared with the normal-sleep group, the disturbed-sleep group exhibited higher scores on all seven PSQI sub-scales (all *p* < 0.01, Table 1); Cohen’s d ranged 0.81–1.36, indicating large practical effects. They also reported significantly elevated tension, anger, fatigue, depression, confusion, self-esteem and total mood disturbance (TMD) (all *p* < 0.01, d = 0.66–1.92, Table 1). Vigor did not differ between groups (*p* = 0.718).

**Table 1:** Participant baseline characteristics, PSQI and POMS scores.

Variable	Disturbed-sleep group (*N* = 87)	Normal-sleep group (*N* = 88)	*p*	*t*
Sex (male / female)	32/55	35/53		
Age (yr)	22.3 ± 3.6	22.9 ± 3.9	0.287	−1.066
Training years (yr)	7.6 ± 2.6	7.7 ± 3.2	0.828	−0.218
Tension (score)	6.50 ± 1.87	4.26 ± 1.92	<0.001	7.532
Anger (score)	7.50 ± 3.20	3.65 ± 1.80	<0.001	9.870
Fatigue (score)	10.2 ± 2.06	3.23 ± 1.55	<0.001	25.154
Depression (score)	5.52 ± 2.12	4.60 ± 1.97	0.004	2.955
Vigor (score)	12.06 ± 2.16	11.95 ± 1.55	0.718	0.361
Confusion (score)	5.80 ± 2.68	4.60 ± 2.10	0.001	3.538
Self-esteem (score)	10.8 ± 2.89	9.50 ± 3.12	0.005	2.867
Total mood disturbance (score)	112.66 ± 6.47	98.89 ± 5.72	<0.001	14.796
PSQI global score	6.97 ± 1.39	2.95 ± 0.89	<0.001	22.585
Subjective sleep quality	0.99 ± 0.42	0.50 ± 0.50	<0.001	6.988
Sleep latency	1.25 ± 0.73	0.49 ± 0.56	<0.001	7.696
Sleep duration	1.82 ± 0.62	1.05 ± 0.69	<0.001	7.753
Habitual sleep efficiency	0.98 ± 0.91	0.14 ± 0.37	<0.001	7.965
Sleep disturbances	0.84 ± 0.43	0.35 ± 0.48	<0.001	7.076
Use of sleep medication	0.07 ± 0.25	0.00 ± 0.00	0.012	2.539
Daytime dysfunction	1.02 ± 0.76	0.43 ± 0.58	<0.001	5.758

Pearson correlations showed that PSQI global score was positively associated with fatigue (*r* = 0.242, *p* = 0.001) and TMD (*r* = 0.347, *p* < 0.001); no other mood dimensions reached significance (Table 2). A-priori forced-entry multiple linear regression with PSQI global score as the dependent variable entered fatigue and TMD simultaneously. The model explained 57% of the variance (adjusted R^2^ = 0.57, *F*(2,172) = 115.59, *p* < 0.001). Fatigue was the strongest predictor (*β* = 0.581, *p* < 0.001), followed by TMD (*β* = 0.218, *p* = 0.004). Variance-inflation factors were < 1.5, ruling out multicollinearity. The regression constant (−3.171) is mathematically necessary but yields negative PSQI predictions only when fatigue and TMD are simultaneously zero—values outside the observed range; within-data predictions remain valid (Tables 3, 4).

**Table 2:** Correlations between PSQI global score and POMS dimensions (*n* = 175).

Variable		Tension	Anger	Fatigue	Depression	Vigor	Confusion	Self-esteem	Total mood disturbance
PSQI	R	0.109	0.031	0.242	0.097	0.085	0.109	0.071	0.347
	P	0.152	0.683	0.001	0.204	0.263	0.152	0.355	0.000

**Table 3:** ANOVA summary for the regression model.

Model		Sum of squares	df	Mean square	*F*	Sig.
1	Regression	540.454	2	270.227	115.595	0.000^a^
	Residual	402.083	172	2.338		
	Total	942.537	175			

Predictors: (Constant), Fatigue, Total mood disturbance.

**Table 4:** Regression coefficients.

Model	Unstandardized Coefficients		Standardized Coefficients	*t*	Sig.
	B	Std. Error	Beta		
(Constant)	−3.171	1.778		−1.783	0.076
Fatigue	0.345	0.044	0.581	7.830	0.000
Total mood disturbance	0.055	0.019	0.218	2.936	0.004

Dependent variable: PSQI score.

Sex-stratified analysis revealed significant gender differences in the association between sleep quality and mood states (Table 5). Correlation analysis showed that the correlation strengths of fatigue, TMD, depression, and tension with PSQI global score were significantly higher in female athletes than in male athletes (all *p* < 0.05). The correlation coefficient between fatigue and PSQI global score was 0.368 (*p* < 0.001) in females and 0.286 (*p* = 0.021) in males. Regression analysis indicated gender differences in the predictive effects of fatigue and TMD on sleep quality: among male athletes, the two variables jointly explained 32% of the variance in PSQI global score (adjusted R^2^ = 0.32, *F* = 18.76, *p* < 0.001), with fatigue as the primary predictor (*β* = 0.512, *p* < 0.001); among female athletes, the predictive model had stronger explanatory power, accounting for 41% of the variance (adjusted R^2^ = 0.41, *F* = 37.82, *p* < 0.001), and both fatigue (*β* = 0.603, *p* < 0.001) and TMD (*β* = 0.257, *p* = 0.001) had higher predictive coefficients than those in males. Intra-group score comparison showed that depression scores in the disturbed-sleep group were significantly higher in female athletes (6.12 ± 2.08) than in male athletes (5.03 ± 2.15, *p* = 0.008); however, there was no significant gender difference in depression scores in the normal-sleep group (*p* = 0.217), suggesting that sleep disturbance has a more prominent impact on depressive mood in female athletes.

**Table 5:** Correlation and regression analysis results between PSQI and POMS dimensions in athletes by gender.

Indicator	Male athletes (*n* = 67)	Female athletes (*n* = 108)	Gender difference comparison (*p*)
1. Correlation Analysis (R/P)			
- Fatigue vs. PSQI Global Score	R = 0.286 / *p* = 0.021	R = 0.368 / *p* < 0.001	0.035
- TMD vs. PSQI Global Score	R = 0.312 / *p* = 0.010	R = 0.402 / *p* < 0.001	0.028
- Depression vs. PSQI Global Score	R = 0.153 / *p* = 0.237	R = 0.296 / *p* = 0.002	0.019
- Tension vs. PSQI Global Score	R = 0.128 / *p* = 0.315	R = 0.214 / *p* = 0.024	0.042
2. Regression Analysis Results			
- Adjusted R^2^	0.32	0.41	-
- *F*-value (df)	18.76 (264) / *p* < 0.001	37.82 (2,105) / *p* < 0.001	-
- Fatigue (Β/P)	Β = 0.512 / *p* < 0.001	Β = 0.603 / *p* < 0.001	-
- TMD (Β/P)	Β = 0.189 / *p* = 0.036	Β = 0.257 / *p* = 0.001	-
3. Intra-Group Score Comparison			
- Depression Score (Disturbed-Sleep Group)	5.03 ± 2.15	6.12 ± 2.08	0.008
- Depression Score (Normal-Sleep Group)	4.82 ± 2.01	4.35 ± 1.89	0.217

## Discussion

Drawing on 175 elite adult athletes from Sichuan Province, China, this study confirms a robust association between sleep quality and mood state. Athletes with disturbed sleep not only displayed elevated global PSQI scores but also impairment across all seven PSQI components, indicating multidimensional sleep deficits. Concurrently, they reported higher levels of tension, anger, fatigue, depression, confusion, self-esteem and TMD, suggesting that poor sleep is accompanied by broad emotional distress. And TMD as predictors, with fatigue accounting for the greatest proportion of variance (*β* = 0.345). These findings partially support our hypothesis that negative affect is positively related to sleep disturbance, with the effect concentrated in the dimensions of fatigue and overall mood disturbance.

Sustained attention during training, poor sleep and fatigue are well-established constraints on elite performance (24, 25); our results are consistent with this view. Existing psychological interventions have typically centered on the training, competition or post-injury phases (26–28). The present data indicate that intervention should be shifted upstream to the management of negative affect, so that sleep quality can be improved in parallel (29, 30). Previous studies have likewise shown that athletes who sleep well report lower tension, fatigue, depression and TMD before both training and competition (31, 32); others have observed differences confined to the confusion sub-scale (33), possibly reflecting variation in sport, sample characteristics or training phase. Difficulty initiating or maintaining sleep, frequent awakenings and vivid dreams erode next-day focus, amplify negative affect and ultimately impair performance.

Our findings align with Brandt et al. (31), who reported that Brazilian elite athletes with poor sleep quality exhibited higher tension, fatigue, and TMD during competitive periods. For endurance athletes, in particular, the strong association between fatigue and sleep disturbance may be attributed to prolonged aerobic training-induced physiological and mental exhaustion (12). In contrast, skill-based athletes (e.g., gymnastics, fencing) showed similar but less pronounced correlations, possibly due to lower training volume but higher psychological pressure from technical precision requirements. Additionally, our results complement Lin et al.’s observation that emotional distress and sleep loss form a bidirectional loop, emphasizing that for team sport athletes, interpersonal stress during training and competition may further amplify this cycle compared to individual sport athletes (16).

Our data further highlight fatigue as the dominant contributor to sleep disturbance. Although elite training involves dual-task motor-cognitive demands that have been hypothesized to induce mental fatigue via transient hypo-frontality (34–37), this mechanism was not directly assessed here; our data simply identify fatigue as the primary psychological predictor of sleep disturbance. Practically, sport psychology staff can therefore target fatigue-related psychological states through structured mental training, thereby improving sleep. Practical, evidence-based extensions include sleep-hygiene education, athlete-adapted cognitive-behavioral therapy for insomnia (CBT-I), and integrated recovery protocols (12). Unexpectedly, self-esteem was slightly higher in the disturbed-sleep group. This may reflect defensive responding under stress or increased self-focus when facing performance deficits (38); alternatively, it could be a chance finding given multiple comparisons.

The gender differences found in this study are consistent with previous research (32). The stronger mood-sleep association in female athletes may be related to dual physiological and psychological factors: physiologically, fluctuations in female hormone levels (e.g., estrogen) may enhance emotional sensitivity, thereby amplifying the interaction between sleep disturbance and depression (16); psychologically, elite female athletes face more prominent gender stereotypes, competitive pressure, and role conflicts (e.g., balancing training and social expectations), leading to a more pronounced impact of negative emotions on sleep (17). In addition, the significantly higher depression scores in females in the disturbed-sleep group suggest that female athletes may be a key target group for sleep-related psychological interventions, requiring tailored support programs.

Limitations should be noted. The cross-sectional design precludes causal inference, and the sample was restricted to 175 elite athletes from a single Chinese province; future work should expand across regions and competitive levels. Training phase (preparation, competition, transition) was not differentiated, yet phase-related mood fluctuation may moderate the emotion–sleep relationship and warrants investigation. Finally, objective sleep indices (polysomnography, actigraphy) were absent; subsequent studies should integrate these to validate subjective PSQI findings against objective sleep architecture.

## Conclusion

In this study of 175 elite adult athletes from Sichuan Province, disturbed sleep was associated with multidimensional sleep deficits across all seven PSQI subscales, as well as psychological fatigue and TMD on the POMS. Tension, anger, depression, and confusion also differed significantly between the disturbed-sleep and normal-sleep groups, but only fatigue and TMD emerged as independent predictors of poor sleep quality. Significant gender differences exist: the associations between fatigue, TMD, and sleep quality are stronger in female athletes, and sleep disturbance has a more prominent impact on their depressive mood. Future interventions should prioritize addressing fatigue and provide gender-specific psychological support for female athletes to improve the sleep quality and mental health of elite athletes.

## Data Availability

The raw data supporting the conclusions of this article will be made available by the authors, without undue reservation.
